# Guidance on the Management of Asymptomatic Blood Donors Who Test Positive for *Babesia*

**DOI:** 10.1093/cid/ciaf721

**Published:** 2025-12-26

**Authors:** Evan M Bloch, Jeremy W Jacobs, Edouard Vannier, Gary P Wormser, Jeffrey A Gelfand, Vijay K Sikand, Howard M Heller, Sunil K Sood, Laura Kirkman, Jennie E Johnson, Luis A Marcos, Jacob E Lemieux, Robert P Smith, Ann E Woolley, Whitney A Perry, Philip M Polgreen, Susan E Beekmann, David J Sullivan, Paul G Auwaerter, Peter J Krause

**Affiliations:** Department of Pathology, Johns Hopkins University School of Medicine, Baltimore, Maryland, USA; Department of Pathology, Microbiology, and Immunology, Vanderbilt University, Nashville, Tennessee, USA; Division of Geographic Medicine and Infectious Diseases, Tufts Medical Center, Boston, Massachusetts, USA; Division of Infectious Diseases, New York Medical College, Valhalla, New York, USA; Division of Infectious Diseases, Department of Medicine, Massachusetts General Hospital, Boston, Massachusetts, USA; Department of Medicine, Tufts University School of Medicine, Boston, Massachusetts, USA; Department of Medicine, Lawrence and Memorial Hospital, New London, Connecticut, USA; Division of Infectious Diseases, Department of Medicine, Massachusetts General Hospital, Boston, Massachusetts, USA; Department of Medicine, Harvard Medical School, Boston, Massachusetts, USA; Northwell Cohen Children's, Infectious Diseases, Zucker School of Medicine, New Hyde Park, New York, USA; Department of Microbiology & Immunology, Weill Cornell Medicine, New York, New York, USA; Department of Medicine, Division of Infectious Diseases, Weill Cornell Medicine, New York, New York, USA; Division of Infectious Diseases, Department of Medicine, Warren Alpert Medical School, Brown University, Providence, Rhode Island, USA; Department of Medicine, Division of Infectious Diseases, Department of Microbiology and Immunology, Stony Brook University, Stony Brook, New York, USA; Division of Infectious Diseases, Department of Medicine, Massachusetts General Hospital, Boston, Massachusetts, USA; Infectious Disease and Microbiome Program, Broad Institute of MIT and Harvard, Cambridge, Massachusetts, USA; Maine Medical Center, Maine Health Institute for Research, Scarborough, Maine, USA; Department of Medicine, Harvard Medical School, Boston, Massachusetts, USA; Division of Infectious Diseases, Brigham and Women's Hospital, Boston, Massachusetts, USA; Division of Geographic Medicine and Infectious Diseases, Tufts Medical Center, Boston, Massachusetts, USA; Department of Internal Medicine, University of Iowa Carver College of Medicine, Iowa City, Iowa, USA; Department of Internal Medicine, University of Iowa Carver College of Medicine, Iowa City, Iowa, USA; Departments of Molecular Microbiology and Immunology, Johns Hopkins Bloomberg School of Public Health, Baltimore, Maryland, USA; Sherrilyn and Ken Fisher Center for Environmental Infectious Diseases, Johns Hopkins University School of Medicine, Baltimore, Maryland, USA; Department of Epidemiology of Microbial Diseases, Yale School of Public Health, New Haven, Connecticut, USA

**Keywords:** Babesia; blood donors; screening; babesiosis; guideline

## Abstract

**Background:**

Regional blood donor screening for *Babesia* has been conducted in the United States since 2019, using highly sensitive and specific nucleic acid testing (NAT). Currently, there are no recommendations regarding the management of asymptomatic blood donors who test positive for *Babesia*.

**Methods:**

A multidisciplinary expert panel was convened to develop guidance for the management of asymptomatic *Babesia*-infected blood donors. Additionally, a survey was distributed through the Infectious Diseases Society of America (IDSA) Emerging Infections Network (EIN) to evaluate how a geographically diverse group of infectious diseases specialists would approach this problem.

**Results:**

The expert panel recommends that all *Babesia* NAT-positive blood donors should be referred for clinical evaluation and retesting using peripheral blood smear (PBS) and *Babesia* PCR. The panel also recommends observation rather than treatment for a reactive molecular test alone. Antimicrobial therapy should be considered for PBS-positive cases. Donors should be instructed to seek medical care if symptoms develop. The EIN survey results are consistent with these recommendations.

**Conclusions:**

Since blood donors comprise healthy, immunocompetent adults, findings of incidental *Babesia* infections in asymptomatic donors are frequently self-limited. Longitudinal studies show that molecular evidence of infection in blood donors clears in almost all without intervention.

## Teaser

Molecular testing of blood donors for *Babesia* is routinely performed in endemic areas of the United States at time of blood donation. This article provides expert guidance on managing asymptomatic blood donors who test positive for *Babesia.*

*Babesia* is a genus of tick-borne intraerythrocytic protozoa that cause babesiosis. Of the *Babesia* species known to infect humans, *Babesia microti* is overwhelmingly predominant in North America [1, 2]. *B. microti* is endemic in the Northeastern and upper Midwestern United States (US). It is readily transmissible via transfusion of any red blood cell-containing blood product. Transfusion-transmitted babesiosis (TTB) is associated with significant morbidity and mortality (up to 19%) [3–5], primarily due to the overrepresentation of individuals with risk factors for severe babesiosis (eg asplenia, hereditary blood disorders such as sickle cell disease, extremes of age, malignancy, HIV/AIDS, immunosuppressive therapy) among transfusion recipients [3, 6]. Prior to routine blood donor screening in the US, over 200 cases of TTB had been reported and >98% were due to *B. microti* [3, 4].

In 2019, blood donor screening for *Babesia* was adopted in 14 US states and Washington, DC where approximately 99% of clinical cases and 95% of TTB cases have been reported [7]. Screening is performed using US Food and Drug Administration (FDA) approved nucleic acid tests (NAT). In the first year of screening, 365 NAT positive donations were identified at the American Red Cross alone, accounting for a large proportion of, albeit not all, donations in *Babesia* endemic areas [8]. Since the initiation of donor testing, cases of TTB have become rare and are largely ascribed to blood donations in states where routine NAT screening is not required [9, 10]. To date, only 1 case of possible NAT failure with subsequent TTB has been reported [11]. Blood donors must be in good health at the time of donation. During a screening interview, they are asked about any medical conditions, along with history of—and/or risk factors for— the major transfusion-transmissible infections, that would preclude donation. Donors undergo a limited physical examination and must also meet minimum hemoglobin thresholds for donation.

There are currently 2 highly sensitive and specific FDA-approved assays in use for blood donor screening for *Babesia*. The Procleix *Babesia* Assay (Grifols Diagnostic Solutions) employs transcription-mediated amplification (TMA) on 0.9 mL of whole blood, which has a limit of detection of 3.0 parasites/mL for *B. microti* [12, 13]. The Cobas Babesia (Roche Molecular Systems), which utilizes 1.1 mL of whole blood, is a qualitative polymerase chain reaction (PCR) assay for *Babesia* DNA and RNA [14, 15]. It has a limit of detection of 2.8 parasites/mL for *B. microti* and a reported specificity of 99.99% or 100% when performed on an individual sample or on pools of 6 donations, respectively [14]. These blood donor NAT assays are 1000-fold more sensitive than PCR assays that are routinely used for clinical diagnostic testing (Supplementary Table 1) [16, 17]. Both assays detect the 4 main pathogenic species infecting humans: *B. microti*, *Babesia duncani*, *Babesia venatorum*, and *Babesia divergens*. However, donor screening is confined to genus only, whereas speciation may be performed separately.

Donors who test positive by NAT are deferred for a minimum of 2 years following the index donation and require a negative repeat *Babesia* NAT before their reinstatement as blood donors. Blood centers typically inform donors of reactive infectious disease testing within 8 weeks of donation deferral [18]. Standard notification letters also explain the reason for deferral and recommend that the donor seek follow-up evaluation and counseling from a healthcare professional. The notification letter does not offer guidance on monitoring or treatment.

Since blood donors must be healthy to qualify for donation, their optimal management following a positive *Babesia* NAT is unclear. To date, there are no established best practice guidelines from professional societies or regulatory bodies for managing this highly selected population.

## METHODS

A multidisciplinary panel of seventeen US-based experts in infectious diseases (ID), transfusion medicine, and primary care was convened to develop a guidance document for the clinical management of asymptomatic blood donors who screen positive for *Babesia* at donation (Table 1). Expertise was determined based on clinical and/or research experience specific to babesiosis. These experts, who constitute the writing panel, were contacted directly via email and invited to participate. As most cases of babesiosis and blood donor screening occur in the Northeastern and upper Midwestern US [7], these regions were overrepresented. Virtual meetings were used to discuss and develop the recommendations.

**Table 1.** Demographics of the Expert Panel (n = 17)

Variable	No. of Respondents (%)
Clinical practice environment	
Hospital-based	8 (47%)
Outpatient	2 (12%)
Both	7 (41%)
Primary practice setting	
Academic	15 (88%)
Community	2 (12%)
Position/specialty	
Adult ID	13 (76%)
Pediatric ID	1 (6%)
Both adult and pediatric ID	1 (6%)
Family medicine	2 (12%)
Hospital bed size (n = 15)	
501–750	1 (7%)
751–1000	2 (13%)
>1000	4 (27%)
…	4 (27%)
…	4 (27%)
Patients seen yearly (not *Babesia* specific)	
101–250	4 (24%)
251–500	2 (12%)
501–750	3 (18%)
751–1000	2 (12%)
>1000	3 (18%)
Not sure	2 (12%)
…	1 (6%)
Number of patients with *Babesia* seen in the last year	
0	2 (12%)
1–3	4 (24%)
4–6	4 (24%)
7–10	1 (6%)
11–20	3 (18%)
>20	3 (18%)

In parallel, a survey was distributed by email to members of the Infectious Diseases Society of America (IDSA) Emerging Infections Network (EIN) [19]. Emerging Infections Network is a provider-based sentinel network supported by the US Centers for Disease Control and Prevention. Emerging Infections Network members include ID physicians, other ID healthcare professionals with advanced degrees (eg, PharmD or advanced practice provider), and public health members who work in federal, state, or local public health departments. The EIN listserv disseminated the survey. As a listserv-based Quick Query, it was limited to 6 questions. The EIN questions spanned testing, treatment, and follow-up. The survey findings were analyzed descriptively. The multidisciplinary panel reconvened to review the survey results and formulate consensus recommendations. The latter were supported by a focused literature review through PubMed and Google Scholar using relevant search terms alone or in combination. Examples of search terms included —but were not limited to— “*Babesia*,” “*Babesia microti*,” “babesiosis,” “blood donors,” “blood donation,” “blood transfusion,” “PCR,” “nucleic acid test,” “peripheral blood smear” and “serology.” The institutional review boards at Johns Hopkins University School of Medicine and Vanderbilt University approved this study.

For this study, “NAT” refers to molecular assays such as PCR and TMA that are used specifically for blood donor screening, whereas “diagnostic PCR” refers to molecular assays that are used in clinical settings (eg follow-up testing of blood donors who screen positive by NAT for *Babesia*). We acknowledge that this distinction is imperfect.

## RESULTS

### Referral

Ideally, cases should be referred to ID specialists with *Babesia* expertise. However, the group recognizes that this is unlikely to occur in many instances and that providers may not have encountered patients with babesiosis.

### Initial Physician Visit

It is recommended that the physician elicits a thorough clinical history, including potential tick exposure, and performs a physical examination, particularly since substantial time is expected to have elapsed between the index donation and the follow-up consultation (Tables 2, 3)

**Table 2.** Responses From the Expert Panel (n = 17)

Survey Question and Denominator	Response Option	N (%)
Next step in management of an asymptomatic, immunocompetent donor with a positive NAT (N = 17)	Retest the donor	11 (65%)
	Clinical observation only	4 (24%)
	Treat the donor	2 (12%)
Timing of retest (only those who chose “retest,” n = 11)	Immediately	10 (91%)
	In 6 m	1 (9%)
	In 3 m	0 (0%)
	In 9 m	0 (0%)
Preferred retest method (n = 11)	NAT (eg PCR) + peripheral blood smear	7 (64%)
	NAT (eg PCR) only	2 (18%)
	Peripheral blood smear only	1 (9%)
	NAT (eg PCR) + peripheral blood smear + serology (IFA)	1 (9%)
Management if repeat test is negative (n = 11)	Observation only	10 (91%)
	Treat donor	1 (9%)
	Retest by a different method	0 (0%)
Management if repeat test is positive (n = 11)	Observation only	4 (36%)^a^
	Treat donor	4 (36%)^b^
	Retest again later	2 (18%)
	Unsure	1 (9%)

^a^Of these four, 2 selected to retest by NAT only, 1 selected both NAT and peripheral blood smear, and 1 selected NAT, serology, and peripheral smear.

^b^Of these four, 3 selected to retest by both NAT and peripheral smear, and 1 selected peripheral smear only.

**Table 3.** Clinical recommendations

Target population	Asymptomatic, immunocompetent, otherwise healthy blood donors who are referred to a healthcare provider for a positive NAT for *Babesia* following a blood donation
Management	Perform a clinical history and physical examination If there are signs or symptoms of infection, or laboratory evidence of hemolysis, treat the individual per the 2020 IDSA babesiosis guideline [25] Retest the blood donor, ideally by molecular assay (PCR for *B. microti*) and peripheral blood smear (PBS) examination If the PBS is positive, obtain a complete blood count Treatment may be considered based on clinical evaluation and the laboratory findings (eg anemia); treatment, when undertaken, should follow the IDSA guideline [25]. Shared decision-making is recommended Counseling and observation may be appropriate If the PBS is negative, treatment is not recommended

### Clinical History

Approximately 20% of *Babesia* infections in immunocompetent adults are asymptomatic and self-limiting [20]. Asymptomatic, persistent parasitemia is well described, in some cases persisting for more than 2 years following initial identification [21]. Healthcare providers should inquire about symptoms and signs of babesiosis, even though donors may not recall the infection and are unlikely to have risk factors for severe babesiosis (eg, HIV/AIDS, cancer, immunosuppressive medications), as these would preclude blood donation. Nonetheless, it is important to ask if the individual failed to disclose any risk factor on the donor history questionnaire or has become immunocompromised since blood donation.

The panel recommended that a health care worker should ask about any obvious tick exposure or history of tick bites, acknowledging that these are unreliable predictors of infection. Less than half (46%) of patients who are diagnosed with babesiosis recall being bitten by a tick [22]. In the US, high proportion of babesiosis cases are transmitted by nymphal stage *Ixodes scapularis* (hard-bodied black-legged) ticks, which are very small and often go unnoticed [23, 24]. *Ixodes scapularis* ticks, which transmit *B. microti*, also transmit *Borrelia burgdorferi* (Lyme disease), *Anaplasma phagocytophilum* (anaplasmosis), *Borrelia miyamotoi* (hard-tick relapsing fever), Powassan virus, *Borrelia mayonii* (Lyme disease), and *Ehrlichia muris eauclairensis* (ehrlichiosis). Healthcare workers should consider the possibility of coinfection, especially Lyme disease, and evaluate for suggestive symptoms and signs. These include rashes (ie, erythema migrans), arthralgia, and neurological symptoms, such as facial palsy.

### Repeat Testing for *Babesia*

**Figure 1.**
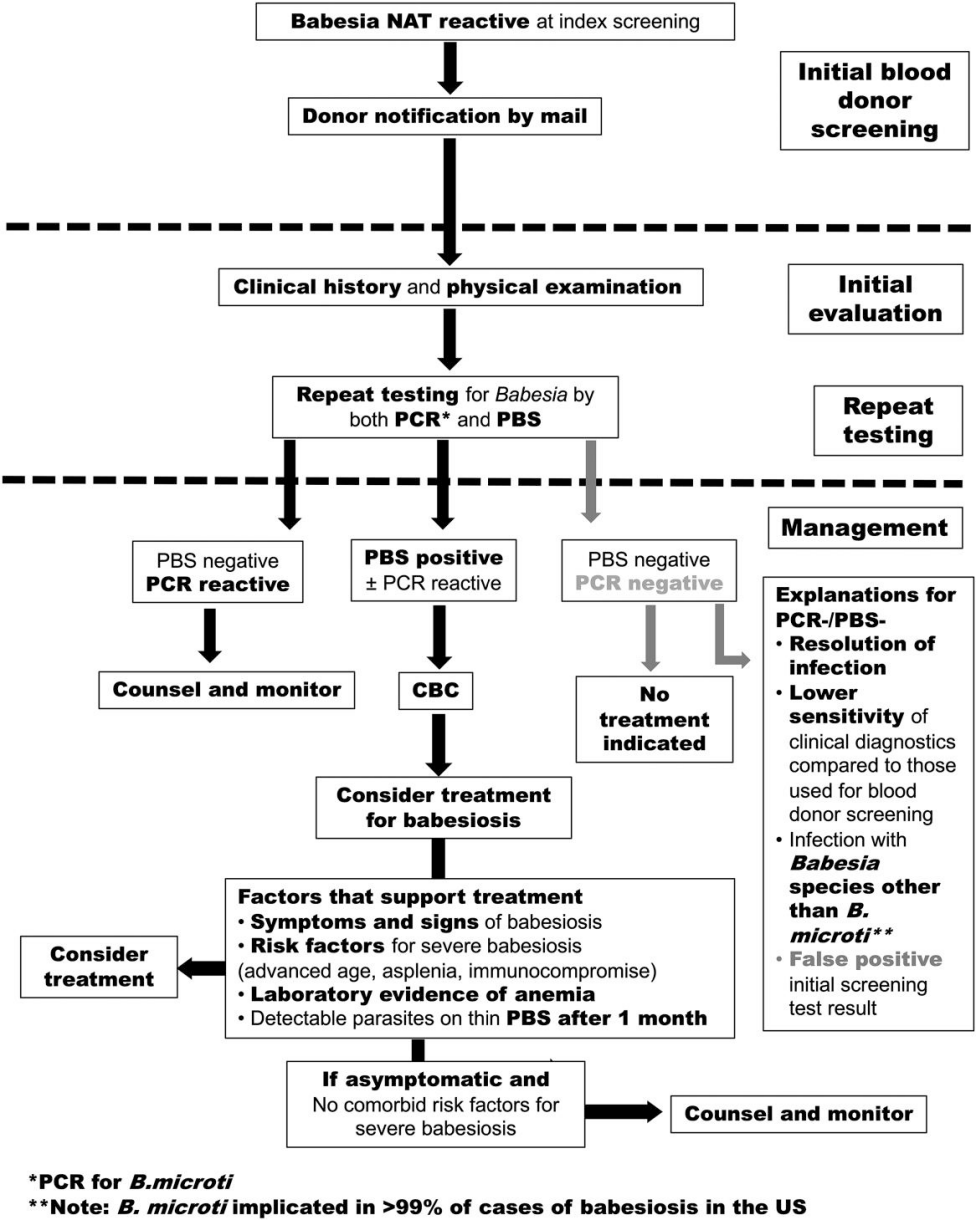
Flow chart of proposed management of blood donor following a positive *Babesia* screening test result.

The panelists recommended obtaining repeat blood testing by both peripheral blood smear (PBS) and diagnostic PCR (for *B. microti*) (Figure 1). However, the availability of such testing varies, which may dictate clinical practice. At a minimum, a repeat PCR for *B. microti* should be performed. There are 4 possible outcomes of repeat testing.

#### Polymerase Chain Reaction Positive/Peripheral Blood Smear Negative

If the PCR is positive but the PBS is negative (the most likely scenario) in asymptomatic individuals, treatment is not recommended [25]. The absence of symptoms and parasites on PBS examination suggests a low parasite burden. PBS has a sensitivity of detection approaching 10–50 parasites/µL (1 infected erythrocyte per 100 000 erythrocytes) with an experienced microscopist. Based on the experience with malaria, the sensitivity of PBS at most diagnostic laboratories is 100–500/µL (or 1 infected erythrocyte per 10 000 erythrocytes) [26]. Clinically available PCR is substantially more sensitive (∼1 parasite/μL or 1 infected erythrocyte per million) than PBS (Supplementary Table 1) [17, 27].

#### Polymerase Chain Reaction Negative/Peripheral Blood Smear Negative

If both PCR and PBS are negative, neither treatment of babesiosis nor follow-up testing are indicated [25]. There are several possible explanations for a non-reactive repeat PCR result. Foremost is the resolution of *Babesia* infection given the interval that will have elapsed since the original positive NAT testing. Approximately half of asymptomatic untreated blood donors clear amplifiable *Babesia* DNA within 4 months, and the majority (86%) clear molecular evidence of infection by 1 year while 95% do so within 2 years [21]. Second, the PCR assays used for clinical diagnostics have a lower sensitivity than NAT used for blood donor screening [17]. Similarly, PBS is relatively insensitive in the setting of low parasitemia, which would be expected in an immunocompetent, asymptomatic blood donor [17]. Third, the clinical PCR assays that target *B. microti* may not detect other human *Babesia* species (Supplementary Table 1).

#### Polymerase Chain Reaction Positive/Peripheral Blood Smear Positive

In the rare event that PCR and PBS are positive, counseling and monitoring alone may still be appropriate, depending on the individual circumstances. The 2020 IDSA Guideline on Diagnosis and Management of Babesiosis does not recommend treatment of asymptomatic *Babesia* infection, unless parasites are detected by PBS for greater than a month [25]. However, in the select case of NAT-positive blood donors, the expected time from donation to notification of the test result, subsequent consultation with a health-care provider and repeat testing is expected to exceed a month (in some cases it could be several months since donation). Therefore, time from donation to clinical follow-up, along with individualized risks (eg anti-*Babesia* drug side effects) and benefits, should be weighed in a shared decision between physician and patient to treat or not to treat.

#### Polymerase Chain Reaction Negative/Peripheral Blood Smear Positive

A PCR-negative/PBS-positive result is theoretically possible if a non-*Babesia microti* species is the cause of infection and the PCR assay in use is specific for *B. microti* (Supplementary Table 1). In the US, almost all (>99%) cases of babesiosis are attributed to *B. microti*.

Factors that may support antimicrobial treatment include symptoms or signs of babesiosis, risk factors for severe babesiosis (that may have developed subsequent to screening or were not acknowledged at the time of donation) and evidence of moderate to severe anemia. A complete blood count is recommended in follow-up to a positive PBS to aid management. If an individual is symptomatic at time of follow-up, management should follow clinical guidelines [25], including consideration of testing for liver inflammation and hemolysis.

If treatment is given to asymptomatic individuals, it should follow the IDSA babesiosis guideline, even though the guideline is largely limited to symptomatic patients with acute or persistent babesiosis [25]. Two combination treatment regimens are recommended in the IDSA guideline: atovaquone plus azithromycin or clindamycin plus quinine. For asymptomatic individuals, we favor atovaquone plus azithromycin because of fewer and less serious adverse events. Consistent with the IDSA guideline, further testing is not indicated for asymptomatic individuals (eg blood donors).

### Additional Considerations

#### Serologic Testing

Serological testing for *Babesia* is not recommended for asymptomatic blood donors; this is consistent with the IDSA guideline [25]. The presence of *Babesia* antibodies may reflect past exposure rather than active infection. Most (92%) individuals remain seropositive for at least 12 months from initial screening despite resolution of the infection [21]. Serologic testing is most commonly performed using an indirect fluorescent antibody test specific for *B. microti*. Low antibody titers can represent false positivity and/or cross-reactivity from pathogens other than *Babesia* [28, 29].

#### Testing for Co-Infection

Several pathogens, most notably *B. burgdorferi* (Lyme disease), are transmitted by the same *Ixodes* tick species that transmits *B. microti*, whereby co-infection is possible [30]. Testing and management for Lyme disease are beyond the scope of this guidance paper. For Lyme disease, professional society guidelines recommend against testing asymptomatic individuals [31].

### Survey of the Emerging Infections Network on the Management of *B. microti* Nucleic Acid Testing-positive Asymptomatic Blood Donors

A total of 167 individuals responded to the EIN survey: 95% (158/167) were ID physicians and 50% (83/167) practiced in a state where routine blood donor screening for *Babesia* is in effect (Table 4). Response patterns were comparable regardless of whether respondents practiced in a state that mandates screening (Supplementary Table 2). Approximately 29% (48/167) of respondents reported treating at least 1 patient with babesiosis in the past year. When asked about the next step in management of an asymptomatic, immunocompetent donor with a positive NAT, 60% (101/167) would retest, 24% (40/167) would elect to observe clinically, and 7% (12/167) would treat. Of respondents who elected to retest, 89% (90/101) would do so either immediately or after 3 months. The preferred methods for re-testing were as follows: PCR 38% (38/101), PBS 37% (37/101) and serology 10% (10/101); 16% (16/101) were unsure. If repeat testing was negative, 79% (80/101) would observe, while 17% (17/101) would retest by a different method. If repeat testing was positive, 56% (57/101) would treat the donor, 26% (26/101) would repeat testing later, and 10% (10/101) would observe only. Stratified by preferred retesting method, 58% (22/38) would treat a donor with a repeat positive result if PCR was used for repeat testing, while 54% (20/37) of respondents who favored PBS for repeat testing would treat if positive.

**Table 4.** Responses to Emerging Infections Network “Quick-Query” on the Management of Asymptomatic Blood Donors With a Positive *Babesia* Nucleic Acid Test (N = 167, Unless Otherwise Noted)

Survey Question and Denominator	Response Option	N (%)
Treated ≥1 patient with babesiosis in the past year (N = 167)	No	114 (68%)
	Yes	48 (29%)
	Not answered	5 (3%)
Primary practice type (N = 167)	Infectious disease (ID) physician	158 (95%)
	Pharmacist (PharmD)	2 (1%)
	Physician other than ID physician	1 (1%)
	Public health professional	1 (0.6%)
	Nurse practitioner	0 (0%)
	Physician assistant	0 (0%)
	Not answered	5 (3%)
Respondents practicing in US states that mandate *Babesia* nucleic acid screening of blood donations (83 of 167 total respondents)	New York	24 (14%)
	Massachusetts	10 (6%)
	Pennsylvania	10 (6%)
	Maryland	9 (5%)
	New Jersey	9 (5%)
	Minnesota	6 (4%)
	Virginia	6 (4%)
	Connecticut	3 (2%)
	New Hampshire	2 (1%)
	Wisconsin	2 (1%)
	District of Columbia	1 (1%)
	Rhode Island	1 (1%)
	Delaware	0 (0%)
	Maine	0 (0%)
	Vermont	0 (0%)
	Other^a^	84 (50%)
Next step in management for an asymptomatic, immunocompetent donor with a positive NAT (N = 167)	Retest the donor	101 (61%)
	Clinical observation only	40 (24%)
	Not sure	14 (8%)
	Treat the donor	12 (7%)
Timing of retest (only those who chose “retest,” n = 101)	Immediately	70 (69%)
	In 3 m	20 (20%)
	Not answered	10 (10%)
	In 6 m	1 (1%)
	In 9 m	0 (0%)
Preferred retest method (n = 101)	NAT (eg PCR)	38 (38%)
	Peripheral blood smear	37 (37%)
	Not sure	16 (16%)
	Serology (IFA)	10 (10%)
Management decisions stratified by preferred retest method (among those selecting “retest”, n = 101)		
Management if repeat NAT (PCR) is negative (n = 38)	Observation only	31 (82%)
	Retest again later	7 (18%)
Management if repeat NAT (PCR) is positive (n = 38)	Treat donor	22 (58%)
	Retest again later	12 (32%)
	Observation only	3 (8%)
	Not answered	1 (3%)
Management if peripheral smear is negative (n = 37)	Observation only	31 (84%)
	Retest again	5 (14%)
	Not answered	1 (3%)
Management if peripheral smear is positive (n = 37)	Treat donor	20 (54%)
	Retest again later	10 (27%)
	Observation only	4 (11%)
	Not answered	3 (8%)

Percentages are those reported in the original quick-query summary and may not sum to exactly 100% because of rounding. Abbreviations: IFA, indirect fluorescent antibody test; NAT, nucleic acid test.

^a^Total number of respondents practicing in the 36 US states where *Babesia* screening is *not* mandated.

## DISCUSSION

This multidisciplinary expert panel and EIN survey results provide the first comprehensive guidance for managing asymptomatic blood donors who test positive for *Babesia* during routine blood donor screening. Our consensus recommendations emphasize clinical evaluation and retesting rather than empirical treatment, reflecting both the unique characteristics of the blood donor population and the natural history of *Babesia* infection. Combined retesting with both PBS examination and PCR is preferred, given the complementary strengths of these modalities in this context. While PCR offers superior sensitivity, it may detect remnant DNA from dead parasites, whereas a positive PBS provides compelling evidence of an active parasite infection. This dual approach helps distinguish donors who may have developed symptoms attributable to *Babesia* infection requiring treatment from those who can be safely observed. Serology is not advised as a first-line retesting approach as it may reflect past exposure rather than active infection.

Asymptomatic *B. microti* PCR positive donors should be counseled regarding symptoms and risk factors for severe babesiosis and instructed to seek immediate medical evaluation if symptoms develop. If they remain asymptomatic with a negative PBS, the expert panel supports observation rather than treatment despite a subsequent reactive molecular test. Several factors support this approach. First, blood donors are a uniquely healthy population, having passed screening questions and basic physical examination requirements. Second, the majority of asymptomatic and mild *Babesia* infections in immunocompetent adults are self-limiting [21, 32, 33]. Third, molecular evidence of infection clears in most asymptomatic blood donors without intervention, emphasizing the self-limiting nature of many *Babesia* infections [21]. Fourth, the risks of treatment must be considered. The preferred babesiosis therapy, namely atovaquone plus azithromycin, is generally well tolerated but can cause cardiac arrhythmias, diarrhea, nausea, transaminase elevations, headache, and rash [34, 35]. Of note, donors should be counseled that, although they feel healthy at the time of the follow-up visit, they may once again test positive by NAT due to various factors (eg persistent submicroscopic infection, continued risk for re-infection), thereby incurring future blood donation deferral [11].

The group identified challenges that could influence decision-making. Given the regional distribution of *Babesia*, clinical experience varies, and this may contribute to differences in management decisions. Blood centers are widely distributed within endemic states, such that donors may not have ready access to specialist care. Cases may be managed by clinicians with varying levels of expertise. In addition, the timing of donor notification introduces additional complexity. FDA regulations require notification within 8 weeks of a positive test. While most EIN respondents recommended retesting either immediately or at 3 months, “immediate” may realistically mean that weeks or even months will have elapsed from the index blood donation to the time of consultation. Resources also vary substantially across healthcare systems. While the ideal retesting strategy includes both PBS and PCR, access may be lacking, necessitating reliance on a single test modality. Lastly, the expert panel does not support routine treatment but recognizes that an individualized approach is warranted in select cases. Treatment decisions may factor proximity to healthcare services, health literacy, change in immune status, and comorbid risk factors for severe babesiosis.

This guidance paper has limitations. The expert panel, while geographically diverse, was relatively small and overrepresented by providers from *Babesia* endemic regions; however, this overrepresentation ensured that panel members had experience treating babesiosis. The EIN survey, although larger and more geographically diverse, needed to be brief to optimize the response rate. We recognize that, by design, the survey was not meant to capture the nuanced decision-making that clinical scenarios may require. As 1 example, the EIN survey did not allow for multiple responses to the retesting approach; the outcome may have been different had respondents been able to include more than 1 test (eg, both PBS and PCR). Lastly, failure to adhere to treatment or seek appropriate care following notification of a positive NAT result are relevant themes but are not discussed as they fall outside the scope of this study.

In conclusion, this expert consensus provides practical guidance for managing asymptomatic blood donors who test positive for *Babesia* at time of donation, emphasizing clinical evaluation, re-testing for *Babesia* using PBS and PCR, and, in most cases, clinical observation. As screening technologies become increasingly sensitive and versatile, research is needed to interpret the associated findings, in order to guide clinical management (ie notification, counseling, and treatment). The principles outlined here—careful clinical assessment, appropriate confirmatory testing, and risk-stratified management—offer a framework for addressing similar challenges posed by other infectious agents/diseases in transfusion medicine.

## Supplementary Material

ciaf721_Supplementary_Data

## Supplementary Data

Supplementary materials are available at *Clinical Infectious Diseases* online. Consisting of data provided by the authors to benefit the reader, the posted materials are not copyedited and are the sole responsibility of the authors, so questions or comments should be addressed to the corresponding author.
