# Supplementary material for: Guidance on the Management of Asymptomatic Blood Donors Who Test Positive for Babesia
Source: Clin Infect Dis. 2025 Dec 26;83(1):e206–13. doi: 10.1093/cid/ciaf721 (PMC12831819; doi:10.1093/cid/ciaf721)
Supplement: ciaf721_Supplementary_Data [file ciaf721_supplementary_data.docx]

**Supplemental Table 1.** Comparison of clinical and blood donor screening tests for *Babesia*

| **Vendor** | **Test methodology** | **Test details** | **FDA-approved** | **Use** |
| --- | --- | --- | --- | --- |
| Mayo Clinic Laboratories | Real-time PCR | Limit of detection:  *B. microti*: 99-2,670 target copies/mL  *B. duncani*: 302-1,540 target copies/mL  *Babesia* MO-1: 10,700 target copies/mL  *B. divergens*: 5,270 target copies/mL | No | Clinical |
| ARUP | PCR | Distinguishes *B. microti* from *B. duncani*, *B. divergens*, *Babesia*MO-1 and *B. venatorum* | No | Clinical |
| Quest Diagnostics | Real-time PCR | Detects *B.* *microti* | No | Clinical |
| LabCorp | Real-time PCR | Detects *B. microti*. Cross-reactivity may occur with *B. duncani*, *B. divergens* and *Babesia* MO-1 | No | Clinical |
| Procleix Babesia Assay[34] (Grifols Diagnostic Solutions)^†^ | Qualitative detection of RNA for the 18S rRNA gene | Limit of detection (95% detection probability):  *B. microti*: 8.91 target copies/mL  *B. divergens*: 11.58 target copies/mL  *B. duncani*: 6.73 target copies/mL  *B. venatorum*: 12.44 target copies/mL  *B. microti*: 2.98 parasites/mL  *B.* *divergens*: 1.77 parasites/mL  *B.* *duncani*: 3.10 parasites/mL | Yes | Blood donor screening |
| Cobas Babesia[35] (Roche Molecular Systems)^†^ | Qualitative detection of RNA and DNA for the 18S rRNA gene | Limit of detection (95% detection probability):  *B. microti*: 2.8 iRBC/mL  *B. duncani*: 52.0 iRBC/mL  *B. divergens*: 16.3 iRBC/mL  *B. venatorum*: 28.3 iRBC/mL | Yes | Blood donor screening |

^†^The test does not distinguish between species.

**Supplemental Table 2.** Responses to Emerging Infections Network “Quick-Query” on the management of asymptomatic blood donors with a positive *Babesia* nucleic acid test stratified by respondents who practice in a state that mandates testing for blood donors versus those that practice in a state that does not mandate testing

| **Survey question & denominator** | **Mandate=Yes**  **N=83** | **Mandate=No**  **N=84** |
| --- | --- | --- |
| **Next step in management for an asymptomatic, immunocompetent donor with a positive NAT (N = 167)**  Observe  Retest  Treat  Not sure | 25 (30%)  49 (59%)  6 (7%)  3 (4%) | 15 (18%)  52 (62%)  6 (7%)  11 (13%) |
| **Timing of retest (only those who chose “retest”, n = 101)**  Immediately  3 months  6 months  Did not answer | 34 (69%)  8 (16%)  1 (2%)  6 (12%) | 36 (69%)  12 (23%)  0 (0%)  4 (8%) |
| **Preferred retest method (n = 101)**  NAT (e.g.PCR)  Serology  Peripheral smear  Not sure  Did not answer | 21 (43%)  4 (8%)  20 (41%)  4 (8%)  0 (0%) | 17 (33%)  6 (12%)  17 (33%)  12 (23%)  0 (0%) |
| **If repeat test is negative, next step (n = 101)**  Observe  Retest  Did not answer | 43 (88%)  5 (10%)  1 (2%) | 37 (71%)  12 (23%)  3 (6%) |
| **If repeat test is positive, next step (n = 101)**  Observe  Retest later  Treat  Did not answer | 5 (10%)  13 (27%)  27 (55%)  4 (8%) | 5 (10%)  13 (25%)  30 (58%)  4 (8%) |
| **Have you treated a patient with babesiosis in past year? (N = 167)**  No  Yes  Did not answer | 38 (46%)  41 (49%)  4 (5%) | 76 (90%)  7 (8%)  1 (1%) |
| **Practice type (N = 167)**  ID physician  PharmD  Public health  Other  Did not answer | 76 (92%)  1 (1%)  1 (1%)  1 (1%)  4 (5%) | 82 (98%)  1 (1%)  0 (0%)  0 (0%)  1 (1%) |
